# Effect of eligibility for referral following mass screening for severe acute malnutrition using mid-upper arm circumference: A prospective regression discontinuity design

**DOI:** 10.1371/journal.pone.0359729

**Published:** 2026-10-08

**Authors:** Huiyu Hu, Mamadou Ouattara, Mamadou Bountogo, Valentin Boudo, Thierry Ouedraogo, Clarisse Dah, Elodie Lebas, Till Bärnighausen, Benjamin F. Arnold, Kieran S. O’Brien, Thomas M. Lietman, Ali Sié, Catherine E. Oldenburg

**Affiliations:** 1 Francis I Proctor Foundation, University of California, San Francisco, United States of America; 2 Centre de Recherche en Santé de Nouna, Burkina Faso; 3 Heidelberg Institute for Global Health, Heidelberg University, Germany; 4 Department of Global Health and Population, Harvard T.H. Chan School of Public Health, Boston, United States of America; 5 Department of Ophthalmology, University of California, San Francisco, United States of America; 6 Institute for Global Health Sciences, University of California, San Francisco, United States of America; 7 Department of Epidemiology & Biostatistics, University of California, San Francisco, United States of America; Innovative Aid, CANADA

## Abstract

**Background:**

Severe Acute Malnutrition (SAM) is a major cause of morbidity and mortality in children under five. Mid-upper arm circumference (MUAC) is widely used for community screening, but the effect of referral eligibility on subsequent outcomes is uncertain.

**Objective:**

This study assesses the intention-to-treat effect of eligibility for referral to a nutritional program, triggered by MUAC below 11.5 cm, on subsequent nutritional status and all-cause mortality among children in Burkina Faso.

**Methods:**

A prospective regression discontinuity (RD) analysis was nested within the Community Health with Azithromycin Treatment (CHAT) trial, a cluster-randomized controlled trial in Nouna District, Burkina Faso (ClinicalTrials.gov NCT03676764). Children aged 6–59 months were screened during twice-yearly censuses; MUAC <11.5 cm triggered a referral recommendation. Because treatment uptake was unmeasured, the estimand was referral eligibility, not treatment receipt. Outcomes were MUAC at the next census and death before that census. Primary analyses used outcome-specific Imbens–Kalyanaraman bandwidths and mixed-effects models with a child random intercept.

**Results:**

Among 65,554 children, 226 (0.34%) met the referral threshold at least once. The selected bandwidth included 2,901 observations for the MUAC outcome (158 below and 2,743 above the cutoff) and 2,628 observations for mortality (148 below and 2,480 above). The estimated mean difference in MUAC at the cutoff was 0.21 cm (95% CI, −0.22 to 0.65), and the mortality odds ratio was 0.95 (95% CI, 0.03 to 31.37). Most sensitivity analyses were similar, although the widest fixed bandwidth produced discordant and unstable estimates.

**Conclusions:**

Near the 11.5-cm cutoff, we found no evidence that referral eligibility changed next-census MUAC or all-cause mortality. These findings do not evaluate SAM treatment receipt or effectiveness, and the mortality estimate was highly imprecise. Stronger mechanisms may be needed to complete referral and link children to treatment.

## Introduction

Globally, severe acute malnutrition (SAM) is a leading contributor to morbidity and mortality in children under five years of age [1], especially in sub-Saharan Africa and South Asia. In Burkina Faso, malnutrition presents a significant challenge, with data indicating that 18% of children under the age of five are underweight (low weight for age), 9% are wasted (low weight relative to height), and 25% are stunted (low height for age) [2]. In the 2013 guideline for the management of SAM, the World Health Organization (WHO) advised the use of mid-upper arm circumference (MUAC) for community-based screening by community health workers (CHWs) for severe and moderate acute malnutrition in children under five years old, using cut-offs of < 11.5 cm for SAM and < 12.5 cm for moderate acute malnutrition (MAM) [3]. MUAC is a simple, quick, and cost-effective tool that can be used at the community level to screen children for acute malnutrition and facilitate referral to nutritional programs. Its simplicity enables it to be used by CHWs and laypersons after minimal training [4], facilitating widespread screening in resource-limited settings. In many nutritional programs, MUAC has been implemented as the sole criterion for admission and discharge, leading to good rates of recovery and weight gain [5,6]. A previous study provided evidence indicating that CHWs have a high capacity to accurately diagnose SAM using MUAC measurements [7]. However, MUAC and weight-for-height z-score (WHZ) identify partially nonoverlapping groups of children with acute malnutrition. Reliance on MUAC alone may miss some children identified by WHZ, whereas WHZ requires calibrated equipment and more intensive training. Thus, the optimal balance between sensitivity and feasibility remains debated [8,9].

Beyond the widespread adoption of MUAC in community-based management of acute malnutrition (CMAM) programs and the choice of screening criterion, it remains unclear whether CHW-led mass screening and referral for SAM improve nutritional status or survival. Screening identifies children who are eligible for referral, but any effect on health outcomes also depends on successful linkage to and receipt of nutritional care.

In community screening for acute malnutrition, referral eligibility to a nutritional program is determined by whether MUAC falls below 11.5 cm. This threshold creates an opportunity to compare children immediately below and above the cutoff, who are expected to be similar except for referral. In this study, we used a prospective regression discontinuity (RD) design to evaluate the intention-to-treat effect of MUAC-based screening and eligibility for referral to a nutritional program on nutritional status and all-cause mortality, utilizing a prospective RD design nested within a large cluster-randomized controlled trial.

## Methods

**Parent trial design.** The Community Health with Azithromycin Treatment (CHAT) trial was a cluster-randomized trial evaluating twice-yearly mass distribution of a single oral dose of azithromycin compared to placebo for prevention of all-cause mortality in children aged 1–59 months. Complete methods and the primary outcome for the trial have been previously reported [10,11]. In brief, 341 communities in Nouna District, Burkina Faso were 1:1 randomized (171 azithromycin; 170 placebo) to biannual mass azithromycin distribution or placebo to children aged 1–59 months. Randomization was stratified by whether communities fell within an existing Health and Demographic Surveillance Site. The allocation list was generated in R using a masked random seed. A door-to-door enumerative census was conducted every 6 months, during which the vital status of each child was assessed and children received study treatment (azithromycin or placebo). The study ran from August 2019 to February 2023, corresponding to 6 rounds of census and treatment. The trial was registered at ClinicalTrials.gov (NCT03676764).

**Ethics and consent.** The study was reviewed and approved by the Institutional Review Board at the University of California, San Francisco and the Comité d’Ethique pour la Recherche en Santé in Ouagadougou, Burkina Faso. The study was conducted in accordance with the Declaration of Helsinki and applicable national regulations. Written informed consent was obtained from at least one guardian of each child enrolled in the study. Analyses used de-identified data, and only authorized study personnel had access to identifiable study records. Patients or the public were not involved in the design, conduct, or reporting of this research.

**Study setting.** CHAT was conducted in Nouna District, Burkina Faso, a rural and mostly agrarian district in northwestern Burkina Faso near the Mali border. Burkina Faso is in the Sahel, which experiences highly seasonal rainfall from July through October, followed by an annual harvest in November-December. Food insecurity, and thus acute malnutrition, are typically highest during the rainy season, when crops are growing. The rainy season also corresponds with the peak malaria transmission season. The Nouna district has high childhood vaccination coverage, established seasonal malaria chemoprevention implementation, and universal access to health care for children under five.

**Communities and participants.** All communities in Nouna District were eligible for inclusion in the trial. After randomization, several communities were lost due to escalating political insecurity occurring in the study area [11]. Inaccessibility occurred at the community level and was not determined by an individual child’s MUAC. All children in the eligible age range (1–59 months) were eligible for treatment during each twice-yearly treatment round. Children were censused up to 65 months of age to evaluate mortality in the 6-month period following each round of treatment administration. Because MUAC-based cutoffs are only validated and used for children ≥6 months of age, this analysis was restricted to children who were 6–59 months of age at the time of first MUAC measurement.

**Running variable and referral assignment.** Trained study census workers measured MUAC during each twice-yearly census. To standardize measurement procedures, workers completed training before each census that included classroom instruction, mock-census exercises, and field-based practice. Census workers used a string to locate the midpoint of the child’s upper arm, and then measured the circumference using a standard MUAC tape. The numeric MUAC measurements were entered into a custom-designed mobile data collection application for the trial. For any recorded MUAC <11.5 cm, the application automatically alerted the worker to recommend referral to a primary health facility for diagnostic confirmation and enrollment in the nutritional program. Because the referral recommendation was generated automatically after the numeric MUAC value was entered, caregivers did not determine either the recorded measurement or application of the referral threshold. However, because the trial could not link census data with health facility records, it did not track whether caregivers sought recommended care.

**Outcomes.** Each eligible MUAC measurement was treated as an index observation. Outcomes were [1] MUAC measured at the next scheduled census, approximately six months later, and [2] all-cause mortality between the index and next census. Children were followed longitudinally up to 65 months of age, although children over 59 months of age did not receive an additional round of treatment. A child could contribute more than one index-follow-up interval. Deaths were included if a child was recorded as alive and resident at the index census and was reported to have died before the next census. Index observations without an observed next-census outcome were excluded from the corresponding complete-case analysis.

### Statistical analysis methods

**Regression Discontinuity Design.** To evaluate the impact of MUAC screening on follow-up MUAC measurements and mortality, we used a sharp RD design, with MUAC as the running variable and a threshold of 11.5 cm for intervention eligibility (referral to the nutritional program). The RD design is a quasi-experimental approach that identifies the causal effects of interventions by exploiting arbitrary threshold rules. Because of noise, children immediately below and above the threshold are expected to have similar underlying prognosis. Thus, in a narrow range around the threshold, exchangeability can be a reasonable assumption, and causal effects can be identified by comparing participants with measurements immediately above and below the threshold. In this study, the sharp RD design allows estimation of the intention-to-treat effect of eligibility for referral among children whose MUAC was near 11.5 cm.

Bandwidth selection in RD involves a bias-variance tradeoff. Wider bandwidths reduce variance by increasing the number of observations but increase bias because they rely on correctly specifying the functional form of the outcome-MUAC relationship. Conversely, narrower bandwidths reduce bias but yield less precise estimates. To balance bias and variance, we implemented the Imbens-Kalyanaraman (IK) algorithm to select optimal MUAC bandwidths for each outcome, improving the precision and validity of the estimates [12]. Additionally, three bandwidths (10.5–12.5 cm, 9.5–13.5 cm, and 8.5–14.5 cm) were used for robustness checks and sensitivity analyses.

**Regression Discontinuity Assumptions.** In our study, assumptions for causal inference in RD were assessed as follows [13,14]. First, assignment (referral) was determined by a known, deterministic cutoff. Second, precise manipulation of the running variable was unlikely: trained workers, rather than caregivers, measured and entered MUAC, and the application automatically generated the referral alert. We examined the frequency of each recorded MUAC value around the cutoff and separately across census rounds (S2 Fig). Recorded MUAC exhibited heaping at rounded values throughout the distribution rather than an isolated accumulation at the 11.5-cm threshold, and similar heaping patterns were observed across census rounds. Third, no other study intervention or service changed at the 11.5-cm cutoff; therefore, potential outcomes in the absence of referral eligibility were assumed to vary smoothly through the threshold.

For the analysis, generalized linear mixed-effects models were utilized, incorporating three different bandwidths selected from IK algorithm. Specifically, let $X_{ij}$ denote index MUAC, c=11.5 cm denote the referral threshold, $E_{ij}=I\left(X_{ij}<c\right)$ indicate eligibility for referral, and $A_{ij}$ denoteindex age in months.The regression model was fitted in the following forms:

1) For next-census MUAC

$Y_{ij}=\beta_{\text{0}}+\beta_{\text{1}}E_{ij}+\beta_{\text{2}}\left(X_{ij}-c\right)+\beta_{\text{3}}E_{ij}\left(X_{ij}-c\right)+\beta_{\text{4}}A_{ij}+u_{i}+\varepsilon_{ij}$

2) For death before the next census

$logit\{\text{Pr}(D_{ij}=\text{1})\}=\beta_{\text{0}}+\beta_{\text{1}}E_{ij}+\beta_{\text{2}}\left(X_{ij}-c\right)+\beta_{\text{3}}E_{ij}\left(X_{ij}-c\right)+\beta_{\text{4}}A_{ij}+u_{i}$

Where$Y_{ij}$ is MUAC at next census, $D_{ij}=\text{1}$ indicates that the child died before the next census, $u_{i}$ is a child-specific random intercept. $\beta_{\text{1}}$is the difference in the cutoff, which is equivalent to the local intention-to-treat effect of eligibility for referral. For the continuous outcome, $\beta_{\text{1}}$ is the adjusted mean difference in next-census MUAC; for mortality, $\text{exp}(\beta_{\text{1}})$ is the corresponding conditional odds ratio. $\beta_{\text{2}}$ is the slope of the line above the threshold, $\beta_{\text{2}}+\beta_{\text{3}}$ is the slope of the line below the threshold and u represents the random effects term, accounting for within-child correlation and the fact that one child could contribute to multiple index–follow-up intervals. We also attempted hierarchical models that included both child- and community-level random intercepts; however, these models did not converge. The final models therefore retained the child-level random intercept only. Because a child's age is associated with mortality and MUAC, we included baseline age in the models to reduce variance. Analyses were completed in R version 4.2.

As a sensitivity analysis, we adjusted for treatment arm (azithromycin vs. placebo), distance to the nearest health facility, and number of children under 5 in the household. We also ran models stratified by treatment arm and included a squared term for the running variable to assess robustness.

## Result

A total of 68,246 children 1–59 months were enrolled in the CHAT study. After excluding 2,692 children who were younger than 6 months at eligible screening observations or had a physiologically implausible MUAC <6 cm, 65,554 children aged 6–59 months remained. Of those, 226 (0.34%) children had at least one recorded MUAC below 11.5 cm and were eligible for referral during the CHAT study period (S1 Fig). This percentage is the cumulative proportion of unique children who ever crossed the referral threshold during repeated population-based censuses, not a cross-sectional prevalence estimate.

Using the IK algorithm, we identified optimal bandwidths for each outcome. For MUAC at follow-up, the optimal bandwidth was 0.70 cm (10.80 to 12.20 cm) and included 158 observations below the cutoff and eligible for referral and 2,743 above the cutoff. For follow-up mortality, the optimal bandwidth was 0.62 cm (10.88 to 12.12 cm), with 148 observations below the cutoff and 2,480 above the cutoff. Characteristics within these bandwidths are shown in Table 1. Recorded MUAC values exhibited substantial heaping at rounded values across the distribution, with similar patterns observed across census rounds rather than a pattern unique to the 11.5-cm referral threshold (S2 Fig).

**Table 1:** Characteristics of observations within the Imbens–Kalyanaraman-selected bandwidths: Characteristics are presented separately for the outcome-specific bandwidths used in the primary regression discontinuity analyses. Data are presented as mean (SD) or n (%), as appropriate.

	10.80 - 12.20 cm ^1^	10.88 - 12.12 cm ^2^
**Number of index observations**	2,901	2,628
**Number of children**	2,654	2,409
**Baseline (index) Age (in month, mean [SD])**	18.7 (12.1)	18.7 (12.2)
**Index MUAC (in cm, mean [SD])**	11.9 (0.3)	11.9 (0.2)
**Number of referral-eligible observation (N [%])**	158 (5.4%)	148 (5.6%)
**Children with ≥1 referral eligible obsertation (N [%])**	156 (5.9%)	146 (6.1%)
**Number of female (N [%])**	1489 (56.1%)	1333 (55.3%)

^1^The optimal bandwidth for Mean MUAC at 6 months, selected using the IK algorithm.

^2^The optimal bandwidth for Mortality at 6 months Odds Ratio selected using the IK algorithm.

In the primary RD analyses, we found no evidence that screening and referral for SAM affected all-cause mortality or nutritional status (MUAC) at 6 months, with results broadly consistent across bandwidths in the sensitivity analysis. Within the IK-selected bandwidth for follow-up MUAC, the estimated mean difference at the cutoff was 0.21 cm (95% CI, −0.22 to 0.65; P = 0.339; Fig 1 and Table 2). Within the IK-selected mortality bandwidth, the odds ratio at the cutoff was 0.95 (95% CI, 0.03 to 31.37; P = 0.978; Table 2). Estimates from the two narrower fixed bandwidths and from covariate-adjusted, treatment-arm-stratified, and quadratic sensitivity analyses were generally consistent with the primary analyses. At the widest fixed bandwidth, however, estimates were discordant and unstable (S1-S4 Tables). All estimates concern eligibility for referral at the cutoff rather than actual receipt of nutritional treatment.

**Table 2:** Regression discontinuity estimates using Imbens–Kalyanaraman-selected bandwidths.

Outcome	Estimate (95% CI)	P-value
**Mean difference in next-census MUAC (cm) ^1^**	0.21 (-0.22 – 0.65)	0.339
**Mortality Odds Ratio (OR) ^2^**	0.95 (0.03 – 31.37)	0.978

^1^The optimal bandwidth for Mean MUAC at 6 months was 0.70 cm (10.80–12.20 cm), selected using the IK algorithm.

^2^The optimal bandwidth for Mortality at 6 months Odds Ratio was 0.62 cm (10.88–12.12 cm), selected using the IK algorithm.

**Fig 1:**
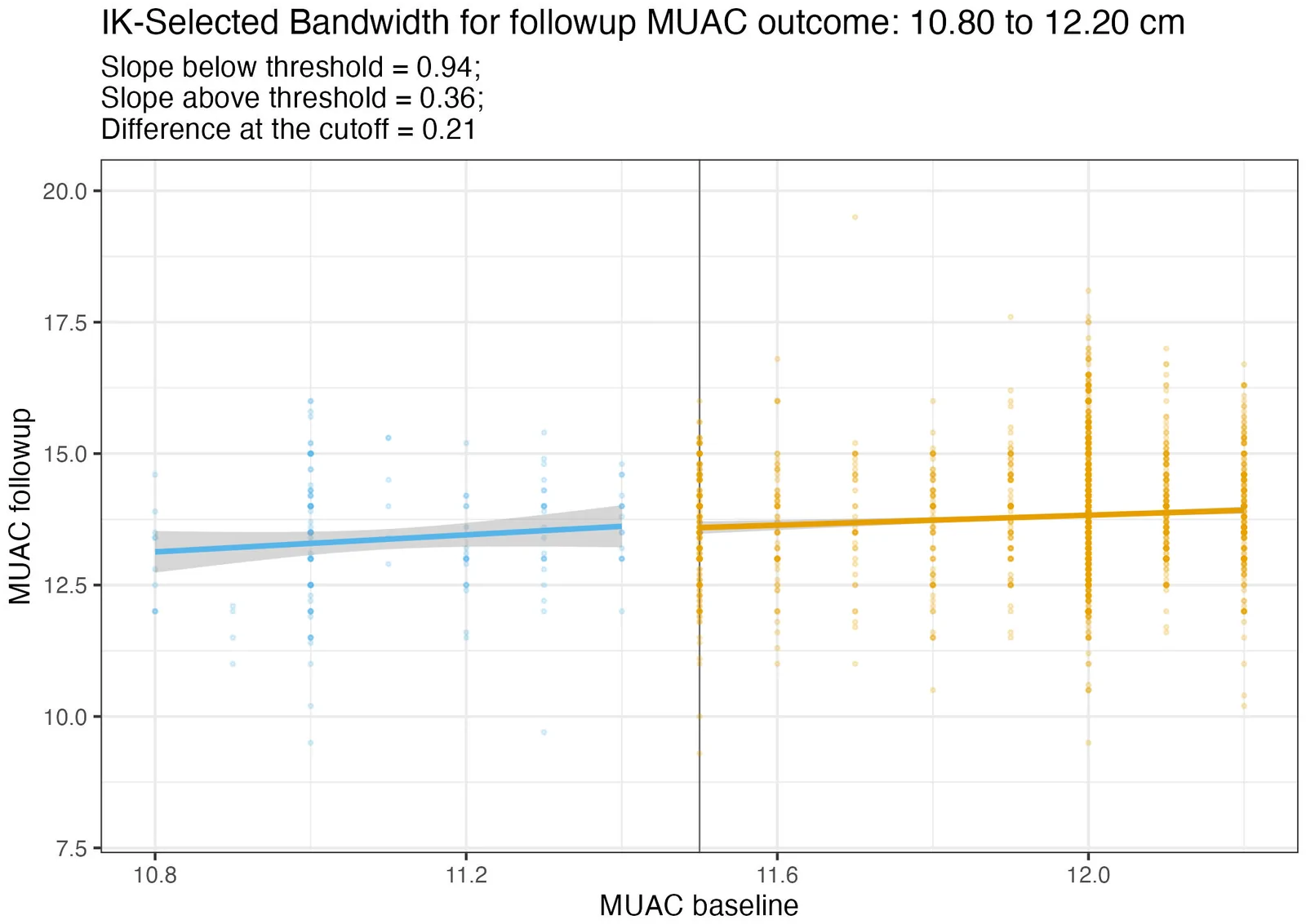
Regression discontinuity estimate for MUAC at the next census. Points represent individual observations within the Imbens–Kalyanaraman-selected bandwidth (10.80 to 12.20 cm). The vertical line indicates the 11.5-cm referral threshold, and fitted lines show the relationship between index MUAC and next-census MUAC on either side of the cutoff, with shaded 95% confidence intervals. The estimated difference at the cutoff was 0.21 cm (95% CI, −0.22 to 0.65).

## Discussion

In this prospective RD analysis nested in a cluster randomized trial, we found no evidence that eligibility for referral following MUAC-based screening changed MUAC at the next census, approximately 6 months later, or reduced all-cause mortality among children near the 11.5-cm referral threshold. The RD estimate represents a local intention-to-treat effect and may not generalize to children with more severe malnutrition. Precision was limited, particularly for the mortality endpoint, as SAM was less common than expected during the study period and mortality is a rare event. Thus, absence of statistical evidence should not be interpreted as evidence of no mortality effect. At the same time, the continuous MUAC outcome was more precisely estimated, but results across bandwidths similarly showed no evidence of improvement. While children with SAM have a higher mortality rate than children without SAM and this endpoint is an important indicator for SAM treatment programs [3,15], programs monitor response to treatment with MUAC, and children who are admitted to nutritional programs are considered to have recovered when their MUAC increases to > 12.5 cm [3]. However, the study did not measure whether referred children accessed nutritional care, and CHAT included no follow-up mechanism to support SAM referral completion. Future studies should evaluate both screening effectiveness and referral completion to improve SAM outcomes.

These findings highlight the importance of connecting children identified with SAM to appropriate care and raise questions about whether screening alone is sufficient to improve SAM outcomes. The effectiveness of MUAC screening depends on downstream referral and treatment pathways. In cluster-randomized studies in Burkina Faso and Mali, substantial increases in acute malnutrition screening did not translate into improved treatment coverage, suggesting important gaps between screening and treatment receipt [16,17]. A previous study estimated that the average coverage of CMAM programs was only 38.3% in sub-Saharan Africa and identified barriers including limited awareness of malnutrition and available programs, as well as geographic and economic barriers to accessing health facilities. [18] In addition, structural barriers, including poverty and lack of access to healthcare can limit caregivers’ ability to follow through with referrals and provide adequate care for their children. For example, an evaluation of a CMAM program in Uganda, caregivers faced significant economic and social constraints that hindered their ability to provide adequate care [19]. Addressing these barriers is therefore essential in settings of extreme poverty, such as rural Burkina Faso.

Potential strategies include integrating treatment of uncomplicated SAM into CHWs’ existing curative responsibilities through integrated community case management, bringing treatment closer to families in rural areas [20]. Programs could also pair screening with active CHW follow-up, home visits or accompaniment after referral, referral-tracking systems with feedback from health facilities, and targeted transportation or financial support for families facing access barriers [21,22]. Training mothers/caregivers to regularly screen for SAM using MUAC presents a cost-effective strategy that enhances coverage, helps with early detection of SAM, and promotes engagement in seeking SAM treatment [23,24].

In addition to strengthening referral pathways, the optimal indicator for detecting acute malnutrition may still require further investigation. MUAC is widely used for SAM screening due to its simplicity and cost-effectiveness. However, ongoing debates in the literature suggest that relying solely on the current WHO-recommended MUAC cutoff, without incorporating weight-for-height (WHZ) as a screening tool, may underestimate severe acute malnutrition cases [8,9]. Yet, WHZ measurement is more complex, requiring calibrated scales and trained personnel, making it impractical for routine use in community-based settings. Future research should explore integrating MUAC with other indicators to improve malnutrition identification while considering the practical constraints of community-based programs.

### Limitations

The study faced several limitations that could impact the interpretation and generalization of findings. First, no formal sample-size calculation was conducted because this secondary analysis included all eligible observations. The small number of referral-eligible observations and rare mortality outcome produced low power and a high risk of Type II error, particularly for mortality; we therefore emphasize confidence intervals rather than statistical significance and assessed robustness across multiple bandwidths and models. Second, the study did not collect data on whether referred children were linked to nutritional programs, leaving gaps in understanding whether those identified through MUAC screening ultimately received care or what their outcomes were. Results of this study should not be interpreted as evaluating the effectiveness of the nutritional programs themselves, but rather of screening and referral to nutritional programs. Third, the study also lacked a mechanism to screen and refer children with moderate acute malnutrition (MAM; MUAC <12.5 cm), preventing us from evaluating outcomes for this important group. While children with MAM are at risk of progressing to SAM and mortality, infrastructure for MAM treatment was not in place during the trial. Fourth, as a large, simple trial, CHAT did not collect some measures, such as WHZ and maternal background such as education, that may have provided additional information on the effect of screening and referral for SAM. Fifth, caregivers could access care independently through weekly nutrition screening at local health facilities, which we did not capture and which may have reduced differences in care between children above and below the referral threshold. Finally, political insecurity caused loss of communities. Because inaccessible communities lacked census data, we could not evaluate systematic differences from retained communities; this limits external validity. Generalizability may also be limited in settings with different distributions of acute malnutrition and child mortality, particularly given the lower-than-expected prevalence of SAM in the study population. These limitations were partly mitigated by standardized worker training, automated application of the referral rule, a child-level random intercept, and sensitivity analyses across bandwidths and model specifications. Further evaluations of the effectiveness of mass screening and referral to nutritional programs are needed, particularly in settings with a higher prevalence of SAM. These results highlight the need for adequately powered evaluations to understand the impact of MUAC-based screening and referral on child health outcomes, including nutrition, mortality, and other relevant indicators.

## Conclusion

Among children near the 11.5-cm MUAC threshold, we found no evidence that eligibility for SAM referral changed MUAC at the next census or all-cause mortality. However, the prevalence of SAM was low, leading to uncertainty in estimates, especially for mortality analyses. The results of this study suggest that screening and referral alone may be insufficient to improve outcomes when linkage to care is incomplete. Future studies should measure the full referral and treatment cascade and be powered to evaluate clinically important nutritional and mortality outcomes.

## Supporting information

S1 Fig Participant flow in the regression discontinuity analyses. The diagram shows the flow of children and index mid-upper arm circumference observations from enrollment in the parent CHAT trial through application of the analysis eligibility criteria and inclusion in the outcome-specific complete-case analyses. Each eligible MUAC measurement was treated as an index observation and linked to MUAC measured at the next scheduled census and vital status before that census. Children could contribute more than one index–follow-up interval.
(DOCX)

S2 Fig Distribution of recorded index mid-upper arm circumference values around the referral threshold. (A) Frequency of recorded index MUAC values from 9.5 to 13.5 cm. The y-axis is presented on a square-root scale to display both common and less frequent measurements. (B) Distribution of recorded index MUAC values from 10.0 to 13.0 cm by census round, expressed as the proportion of observations within each round. Dashed vertical lines indicate the 11.5-cm referral threshold.
(DOCX)

S1 Table Regression discontinuity estimates using fixed bandwidths around the 11.5-cm referral threshold. The table presents the estimated mean difference in MUAC at the next scheduled census and the odds ratio for all-cause mortality before that census, comparing observations eligible for referral because index MUAC was below 11.5 cm with observations not eligible for referral immediately above the threshold. Models used linear functions of centered index MUAC with separate slopes below and above the cutoff, adjusted for index age, and included a child-specific random intercept to account for repeated index–follow-up intervals.
(DOCX)

S2 Table Regression discontinuity estimates stratified by randomized treatment arm. Estimates are presented separately for communities randomized to azithromycin and placebo in the parent CHAT trial. Analyses used the outcome-specific Imbens–Kalyanaraman-selected bandwidths: 10.80–12.20 cm for next-census MUAC and 10.88–12.12 cm for all-cause mortality. Models compared observations eligible for referral because index MUAC was below 11.5 cm with observations not eligible for referral immediately above the threshold, adjusted for index age, and included a child-specific random intercept.
(DOCX)

S3 Table Covariate-adjusted regression discontinuity estimates across outcome-specific and fixed bandwidths. The table presents the estimated mean difference in MUAC at the next scheduled census and the odds ratio for all-cause mortality before that census at the 11.5-cm referral threshold. Models adjusted for index age, distance to the nearest health facility, number of children under five years of age in the household, and randomized treatment arm, and included a child-specific random intercept. The Imbens–Kalyanaraman-selected bandwidths were 10.80 to 12.20 cm for next-census MUAC and 10.88 to 12.12 cm for all-cause mortality.
(DOCX)

S4 Table Regression discontinuity estimates using quadratic functional-form specifications. The table presents sensitivity analyses using quadratic functions of centered index MUAC, with interactions between referral eligibility and the linear and quadratic terms to allow the relationship between index MUAC and each outcome to differ below and above the 11.5-cm cutoff. Models were adjusted for index age and included a child-specific random intercept.
(DOCX)
