# Supplementary material for: Effect of eligibility for referral following mass screening for severe acute malnutrition using mid-upper arm circumference: A prospective regression discontinuity design
Source: PLoS One. 2026 Oct 8;21(10):e0359729. doi: 10.1371/journal.pone.0359729 (PMC13648963; doi:10.1371/journal.pone.0359729)
Supplement: Supplementary file 1 [file pone.0359729.s001.docx]

**S1 Fig. Participant flow in the regression discontinuity analyses.** The diagram shows the flow of children and index mid-upper arm circumference observations from enrollment in the parent CHAT trial through application of the analysis eligibility criteria and inclusion in the outcome-specific complete-case analyses. Each eligible MUAC measurement was treated as an index observation and linked to MUAC measured at the next scheduled census and vital status before that census. Children could contribute more than one index–follow-up interval.
