# Supplementary material for: Effect of eligibility for referral following mass screening for severe acute malnutrition using mid-upper arm circumference: A prospective regression discontinuity design
Source: PLoS One. 2026 Oct 8;21(10):e0359729. doi: 10.1371/journal.pone.0359729 (PMC13648963; doi:10.1371/journal.pone.0359729)
Supplement: Supplementary file 2 [file pone.0359729.s002.docx]

### **S2 Fig. Distribution of recorded index mid-upper arm circumference values around the referral threshold. (A)** Frequency of recorded index MUAC values from 9.5 to 13.5 cm. The y-axis is presented on a square-root scale to display both common and less frequent measurements. **(B)** Distribution of recorded index MUAC values from 10.0 to 13.0 cm by census round, expressed as the proportion of observations within each round. Dashed vertical lines indicate the 11.5-cm referral threshold.
