# Supplementary material for: Effect of eligibility for referral following mass screening for severe acute malnutrition using mid-upper arm circumference: A prospective regression discontinuity design
Source: PLoS One. 2026 Oct 8;21(10):e0359729. doi: 10.1371/journal.pone.0359729 (PMC13648963; doi:10.1371/journal.pone.0359729)
Supplement: Supplementary file 3 [file pone.0359729.s003.docx]

**S1 Table. Regression discontinuity estimates using fixed bandwidths around the 11.5-cm referral threshold.** The table presents the estimated mean difference in MUAC at the next scheduled census and the odds ratio for all-cause mortality before that census, comparing observations eligible for referral because index MUAC was below 11.5 cm with observations not eligible for referral immediately above the threshold. Models used linear functions of centered index MUAC with separate slopes below and above the cutoff, adjusted for index age, and included a child-specific random intercept to account for repeated index–follow-up intervals.

| **Range** | **Mean MUAC at 6 months after screening (95% CI)** | **P-value** | **Mortality Odds Ratio (95% CI)** | **P-value** |
| --- | --- | --- | --- | --- |
| **10.5-12.5 cm** | 0.08 (-0.24 – 0.39) | 0.62 | 0.88 (0.09 – 8.83) | 0.91 |
| **9.5-13.5 cm** | -0.14 (-0.35 – 0.08) | 0.22 | 1.12 (0.24 – 5.22) | 0.89 |
| **8.5-14.5 cm** | -0.22 (-0.42 – -0.02) | 0.03 | 42.54 (1.71 – 1057) | 0.02 |
