# Supplementary material for: Effect of eligibility for referral following mass screening for severe acute malnutrition using mid-upper arm circumference: A prospective regression discontinuity design
Source: PLoS One. 2026 Oct 8;21(10):e0359729. doi: 10.1371/journal.pone.0359729 (PMC13648963; doi:10.1371/journal.pone.0359729)
Supplement: Supplementary file 4 [file pone.0359729.s004.docx]

**S2 Table. Regression discontinuity estimates stratified by randomized treatment arm.** Estimates are presented separately for communities randomized to azithromycin and placebo in the parent CHAT trial. Analyses used the outcome-specific Imbens–Kalyanaraman-selected bandwidths: 10.80–12.20 cm for next-census MUAC and 10.88–12.12 cm for all-cause mortality. Models compared observations eligible for referral because index MUAC was below 11.5 cm with observations not eligible for referral immediately above the threshold, adjusted for index age, and included a child-specific random intercept.

| **Treatment arm** | **Mean MUAC at 6 months after screening (95% CI)** | **P-value** | **Mortality Odds Ratio (95% CI)** | **P-value** |
| --- | --- | --- | --- | --- |
| **Azithromycin** | 0.46 (-0.30 – 1.23) | 0.23 | 0.01 (0.00 – 1.0e+10) | 0.91 |
| **Placebo** | 0.16 (-0.45 – 0.78) | 0.61 | 1.91 (0.03 – 142.99) | 0.77 |
