# Supplementary material for: Effect of eligibility for referral following mass screening for severe acute malnutrition using mid-upper arm circumference: A prospective regression discontinuity design
Source: PLoS One. 2026 Oct 8;21(10):e0359729. doi: 10.1371/journal.pone.0359729 (PMC13648963; doi:10.1371/journal.pone.0359729)
Supplement: Supplementary file 5 [file pone.0359729.s005.docx]

**S3 Table. Covariate-adjusted regression discontinuity estimates across outcome-specific and fixed bandwidths.** The table presents the estimated mean difference in MUAC at the next scheduled census and the odds ratio for all-cause mortality before that census at the 11.5-cm referral threshold. Models adjusted for index age, distance to the nearest health facility, number of children under five years of age in the household, and randomized treatment arm, and included a child-specific random intercept. The Imbens–Kalyanaraman-selected bandwidths were 10.80 to 12.20 cm for next-census MUAC and 10.88 to 12.12 cm for all-cause mortality.

| **Range** | **Mean MUAC at 6 months after screening (95% CI)** | **P-value** | **Mortality Odds Ratio (95% CI)** | **P-value** |
| --- | --- | --- | --- | --- |
| **IK-selected bandwidth** | 0.21 (-0.23 – 0.64) | 0.35 | 1.01 (0.03 – 34.15) | 0.99 |
| **10.5-12.5 cm** | 0.08 (-0.24 – 0.39) | 0.64 | 0.82 (0.08 – 8.40) | 0.87 |
| **9.5-13.5 cm** | -0.11 (-0.33 – 0.11) | 0.31 | 1.10 (0.24 – 5.12) | 0.90 |
| **8.5-14.5 cm** | -0.20 (-0.40 – 0.01) | 0.06 | 0.02 (0.00 – 1.13) | 0.06 |
