# Supplementary material for: Effect of eligibility for referral following mass screening for severe acute malnutrition using mid-upper arm circumference: A prospective regression discontinuity design
Source: PLoS One. 2026 Oct 8;21(10):e0359729. doi: 10.1371/journal.pone.0359729 (PMC13648963; doi:10.1371/journal.pone.0359729)
Supplement: Supplementary file 6 [file pone.0359729.s006.docx]

**S4 Table. Regression discontinuity estimates using quadratic functional-form specifications.** The table presents sensitivity analyses using quadratic functions of centered index MUAC, with interactions between referral eligibility and the linear and quadratic terms to allow the relationship between index MUAC and each outcome to differ below and above the 11.5-cm cutoff. Models were adjusted for index age and included a child-specific random intercept.

| **Range** | **Mean MUAC at 6 months after screening (95% CI)** | **P-value** | **Mortality Odds Ratio (95% CI)** | **P-value** |
| --- | --- | --- | --- | --- |
| **IK-selected bandwidth** | -0.07 (-0.83 – 0.69) | 0.85 | 0.00 (0.00 – 2,485,404) | 0.20 |
| **10.5-12.5 cm** | 0.13 (-0.43 – 0.69) | 0.66 | 0.89 (0.01 – 79.10) | 0.96 |
| **9.5-13.5 cm** | 0.03 (-0.34 – 0.40) | 0.88 | 0.60 (0.04 – 8.93) | 0.71 |
| **8.5-14.5 cm** | 0.13 (-0.20 – 0.46) | 0.43 | 6.12 (0.42 – 88.90) | 0.19 |
